# Quality of Chinese government environmental health information disclosure during COVID-19 pandemic: Satisfaction survey on University students

**DOI:** 10.3389/fpubh.2022.948172

**Published:** 2022-09-23

**Authors:** Ruikun An, Feng Wang, Yihan Hou, Kitagawa Hideki

**Affiliations:** ^1^School of Economics and Management, Northwest University, Xi'an, China; ^2^Faculty of Policy Study, Ryukoku University, Kyoto, Japan

**Keywords:** COVID-19 pandemic, quality of government information disclosure, environmental health knowledge, environmental health awareness, environmental behavior, environmental health knowledge disclosure

## Abstract

Government played a vital role during the COVID-19 pandemic by disclosing related environmental health information to the public. A satisfaction survey is often used to evaluate the public's satisfaction of the government's information disclosure while reflecting problems in the current disclosure system. As University students generally have better cognitive skills, they efficiently received related information during the pandemic, and therefore 717 questionnaires completed by University students were selected for this study. During the pandemic, the quality of the government's environmental health information disclosure system ranked at 13.89, marginally higher than average. Moreover, the timeliness and content adequacy of the disclosure system ranked at a level slightly above average. By adopting Hayes PROCESS Model 4 and 8, this study found that there is a direct impact of environmental health knowledge and environmental health awareness on satisfaction. Furthermore, University students' environmental health knowledge and awareness enhanced satisfaction through the mediating effect of self-reported environmental behavior. Finally, this study attempted to discover the conditions under which environmental health knowledge and awareness would have a greater direct and indirect influence on satisfaction, that is, the reverse moderating effect of household income level. In addition, this paper offers policy recommendations to enhance quality of government environmental health information disclosure system.

## Introduction

Since the outbreak of COVID-19 pandemic in December 2019 in Wuhan, China (1), enormous amount of news, information, and data from mass media about the pandemic flooded the entire nation (2). To this day, the pandemic has yet to be contained completely nationwide; the public has gradually become more aware of environmental information, as well as the health issues and infectious diseases caused by human activities (3). For example, anthropogenic land use, which both directly and indirectly reshaped our ecosystem (4, 5), has increased the interactions between wild animals and humans (6) which has turned gradually into a public health crisis (7). The COVID-19 pandemic as a rare and extreme public health emergency (8) also challenged the Chinese government information disclosure system which has a prominent status within the current environmental regulation system (9).

Among the different types of information that the government disclosed to the public during the COVID-19 pandemic, environmental health information played an essential part. Environmental health information not only reveals environmental issues, but also reveals how certain environmental issues will affect human health. For example, instead of merely disclosing pollutant level, the government would disclose to what extent the pollutant level will severely harm human health. Hence, this paper defines environmental health information disclosure as a procedure of publicizing information related to how certain environmental concerns impact human health to the public. The government's environmental health information disclosure system is an essential component of the current government information disclosure system. Within government environmental health information disclosure, while public participation can be seen as the soul of environmental regulations (10), the right to know of environmental health information acts as the foundation and perquisite of public participation (11). If public participation cannot be implemented, then government information disclosure and environmental policy formulation function cannot be scrutinized by the public (12). Similarly, environmental policy would be ineffective without public participation, and environmental protection will therefore become ineffectual (13). Consequently, environmental information disclosure is a prerequisite to public participation.

The quality of government environmental health information disclosure is often difficult to directly evaluate, and hence research usually measures it with indirect methods such as citizen feedback (14). Satisfaction research is often used to measure the public satisfaction, and it can directly reflect the quality of the current Chinese government environmental health information disclosure as public satisfaction shows the quality of government work to a certain extent (15). Satisfaction is measured by the feeling resulting from comparing between an individuals' perception of a product or service and their previous expectation (16), and when the reality and public expectation diverge, public satisfaction will change correspondingly (17). Accordingly, this study chooses respondents' satisfaction of the government's environmental health information disclosure as citizen feedback to reflect the quality of the government's environmental health information disclosure during the COVID-19 pandemic. As public satisfaction is one of the most important factors to evaluate government service quality, it follows that satisfaction of the government's environmental health information disclosure indicates the quality of the government's environmental health information.

This study chose University students as the research sample, because University students as one of the plural subjects in social governance played a unique and vital role in the prevention and control during the COVID-19 pandemic (18). Due to the rapid transmission and the extremely high risk of COVID-19, and to protect University students' safety while ensuring their education was not affected by the pandemic, the Ministry of Education of the People's Republic of China requested universities to postpone the start date of the semester nationwide, and brought up the governance philosophy of “suspending classes without stopping to teach, suspending classes without stopping to learn” (19). Hence online learning has become an essential method to spread and communicate knowledge for University students. Online learning ensures that not only the knowledge is being communicated substantially, but also demonstrates that University students have more access to information about the COVID-19 pandemic as compared to other social groups due to the significant amount of time they spent online during the pandemic (20). Consequently, it is reasonable to choose University students as the research sample to represent their satisfaction of the government's environmental health information disclosure during COVID-19. Moreover, University students are the main public group of the current knowledge and information era, and their lifestyle and opinion have been changed by this information age, as they are more familiar with the current ways of acquiring information, selecting information, and using information (21). In general, University students have higher abilities in receiving information and cognition, while they received related information efficiently during the pandemic, and hence could objectively reflect the satisfaction of government environmental health information disclosure. Accordingly, this paper chose University students as research subjects to analyze the impact of their environmental health cognition on satisfaction of government's environmental health information disclosure.

After the rapid transmission of COVID-19, many scholars have debated the effectiveness of the different policies and government information disclosure procedures that different countries implemented during the pandemic. From the perspective of epidemiology, researchers examined the effectiveness of non-pharmaceutical interventions (NPIs) that many governments adopted. A previous study demonstrates that NPIs such as lockdown could reduce the transmission effectively (22), and another study suggests that isolation for new confirmed cases highly reduced the transmission as well (23). Moreover, a later study shows that closing schools and universities and restricting gatherings more than 10 people both could effectively reduce the transmission (24). During the pandemic, citizen coproduction has been widely implemented to reduce the rapid transmission of the virus, and it is needed even after COVID-19 (25). From the perspective of the relationship between government and citizen, a previous study suggests that citizen coproduction is positively affected by government information disclosure, while citizens' trust in local government has an impact on the positive effect (26). Furthermore, citizens' environmental concern positively impacts their opinions toward long-term public policies and influences their opinion of the trade-off between the overall economy and the public health (27). From the perspective of open government, the public is generally satisfied with the COVID-19 pandemic information disclosed by the government, but they feel they lack opportunities to participate (28). A previous study also examined the effectiveness of government information disclosure on microblogging platforms during COVID-19 (29). Previous studies further highlight the importance of global governance (30) and necessity of health equity system for the government to be prepared for the future public health emergences (31). Table 1 shows the summary of some related literatures after the COVID-19 pandemic.

**Table 1:** Post COVID-19 pandemic literatures summary.

**References**	**Results and findings**
Flaxman et al. (22)	Lockdown as one common non-pharmaceutical intervention has significant effect on decreasing transmission.
Lai et al. (23)	Non-pharmaceutical intervention such as isolation for new confirmed cases highly reduced the transmission.
Brauner et al. (24)	Non-pharmaceutical interventions such as closing schools and universities and restricting gatherings over 10 people both could effectively reduce transmission.
Steen and Brandsen (25)	Sustainable citizen coproduction is needed after COVID-19 and government could start with building supportive legal systems.
Wu et al. (26)	Government information disclosure boosted citizen coproduction during COVID-19, and citizens' trust in local government moderated this positive effect.
Walker et al. (32)	Strict intervention strategies for low- and middle-income countries are essential before vaccine becomes available.
Escario et al. (27)	Citizens' environmental concern positively impacts their opinions toward long-term public policies and influences their opinion of the trade-off between the overall economy and the public health.
Park et al. (28)	From the perspective of open government, the public is generally satisfied with the COVID-19 pandemic information disclosed by the government, but they consider they lack opportunities to participate.
Zhang et al. (29)	Examined the effectiveness of government information disclosure on microblogging platforms during COVID-19.
Boschele (30)	COVID-19 pandemic is more than a public health emergency, it is also an emergency for global governance.
Feng and Kirkley (33)	Online geolocalized emotion is impressionable to key COVID-19 policy announcements at a national level, and the impact varies between different cities.
Alberti et al. (31)	Proposed a framework for the government to be prepared for future public health emergencies.

Unfortunately, previous studies rarely include environmental health cognition, self-reported environmental behavior, and satisfaction of the government's environmental information disclosure in one context, then evaluate the impact of environmental health knowledge and awareness on satisfaction of the government's environmental health information disclosure during the COVID-19 pandemic. Therefore, this paper collected University students' data through a questionnaire survey to analyze the effect of their environmental health knowledge and awareness on their satisfaction of the government's environmental health information disclosure by applying a simple mediation model and a moderated mediation model.

This paper's contributions are as follows: first, government information disclosure has been proved highly efficient both for the government and the citizens in reducing the transmission of the virus during the COVID-19 pandemic; this paper found the average satisfaction of government environmental health information disclosure during the COVID-19 pandemic for our sample is 13.89, marginally higher than the “generally satisfied” level, indicating the quality of government environmental health information disclosure during the pandemic is relatively poor. It also found that University students' satisfaction of the timeliness and content adequacy of environmental health information disclosure are lower than that of general environmental information disclosure. Second, this paper chose the perspective of University students to analyze their satisfaction during the COVID-19 pandemic, a rare and extreme public health crisis, as they received more related information compared to other social groups which enhanced the objectiveness of this paper, also expanded the current literature on satisfaction of government environmental health information disclosure. Moreover, this paper evaluated the mediating effect of self-reported environmental behavior between environmental health knowledge and awareness and the satisfaction with the government's environmental health information disclosure. Finally, this paper assessed the moderating effect of household income level on the impact of environmental behavior on satisfaction and found that different income levels' self-reported environmental behavior present various degrees of impact on satisfaction of the government's environmental health information disclosure.

This paper contains the following sections: literature review and hypotheses, method, results and analysis, and conclusion and policy recommendations.

## Literature review and hypothesis

### Individuals' environmental knowledge and environmental awareness on environmental behavior

Theory of Planned Behavior (TPB) has been widely applied in individual environmental behavior related research (34). Based on rational behavior theory, the theory states that individuals' environmental behavior is highly affected by their intentions which involves their attitude, control of their behavior, and the subjective standards (35). This indicates that the more positive attitude individuals have toward a certain environmental behavior, the greater subjective pressure they will feel, the more behavior control they will receive, the greater behavioral intention they will develop, and therefore they will act on that certain environmental behavior. Based on the above theory, scholars have developed research on the influential factors of individuals' satisfaction which can be summarized into three main categories: demographic factors, psychological factors, and internal factors (36). From the perspective of psychology, public subjective opinion of the government's environmental health information disclosure contains environmental health knowledge and awareness (37, 38).

Psychological factors such as cognition of the seriousness of environmental problems, environmental concern, environmental emotion, subjective norms, and personality traits have been proven to have an important impact on the implementation of public environmental behavior (39). Many studies are related to environmental behavior, and its influential factors arose from environmental psychology, and they are the psychological motivation of occurrence and continuation of environmental behavior (39–41). Scholars also discovered that other influencing factors, including environmental sensitivity, knowledge of environmental strategies, and attitude of environmental pollution have a significant impact on environmental behavior (42). Research found that environmental knowledge and environmental awareness are the main influencing factors of environmental behavior among younger generations (43). When individuals decide to take certain action, knowledge and awareness always come first, then the behavior follows, with unconscious behavior being the exception. It is believed that knowledge, awareness, and behavior are the “unity of knowledge and action” (44), meaning individuals' environmental health knowledge and awareness lies behind their environmental behavior. Environmental health knowledge is also considered as one vital condition that cause individuals to participate in environmental behavior, and the impact of environmental behavior on environmental knowledge has been discussed in many theoretical models. In the model of environmental behavior, both environmental knowledge and behavior strategy knowledge have a direct connection with environmental behavior (45). According to differential exposure theory, a worse general environment will lead to a situation where more attention will be given to environmental issues from the public (46). As social media and public communication are ways for the public to obtain environmental health information, environmental knowledge becomes a vital factor that could influence individuals' actions after the exposure, and environmental knowledge as a by-product of the delivery of environmental health information presents a positive correlation with environmental behavior in the private sphere (45).

While scholars have defined the concepts of environmental knowledge and environmental awareness in different ways, there are no unified conceptual definitions for environmental health knowledge and environmental health awareness. For example, environmental knowledge refers to system knowledge of the operation of an ecosystem, action-related knowledge of the applicability toward environmental behavior, and effectiveness knowledge on certain environmental behaviors (46). It also refers to general facts and information relating to an environment and ecosystem (47), or knowledge of solutions of environmental issues (48). As for environmental awareness, some research considers it to contain environmental knowledge, environmental values, environmental attitudes, and willingness of actions and behavior (49), while others consider environmental knowledge as one determining factor of environmental awareness (48). Considering the content of this paper, and the concepts of environmental knowledge and environmental awareness, this paper defines as follows: environmental health knowledge is defined as information including environmental knowledge, health knowledge, and the impact of certain environment issues on individuals' health; environmental health awareness contains not only environmental awareness, but also an individual's mentality, sense of identity toward the environment, and the vigilance of the impact of environment on individual's health.

### The mediating effect of environmental behavior

Hines et al. (44) and other scholars define environmental behavior as behavior with a responsible nature, and divide it into persuasive behavior, consumer behavior, ecological management behavior, legal action, and political action based on different types of responsibility. Stern (50) divides environmental behavior into public sphere environmental behavior and private sphere environmental behavior based on the space in which the behavior happens. This paper focuses on self-reported environmental behavior, that is, private sphere environmental behavior. For example, actively sorting household waste, taking public transport instead of driving, and using reusable bags for shopping.

Satisfaction is a subjective definition used to describe the degree of gaining pleasure from an individual's desire, expectation or need. To extend this definition to a conceptual level, with the consideration of the SERVQUAL Model (51–53), satisfaction of the government's environmental health information disclosure can be seen as satisfaction of the government's environmental health information disclosure that meets individuals' expectations. The subject of satisfaction of the government's environmental health information disclosure is individual, the object is the timeliness and the content adequacy of environmental health information disclosure, and the connection between the subject and the object is the individual's perception of the environmental health information disclosure. According to the basic view from psychologists, an individual's perception of the environment influences the behavior (54), which raises the question of what kind of relationship exists between personal environmental behavior and satisfaction.

Public awareness of environmental health develops along with the process of social construction, and their environmental health knowledge and awareness reflect the attitude and value orientation toward environmental issues (55, 56), which could further affect their objective perception and judgment. Studies have shown that there is a correlation between public awareness of environmental issues, participation in pro-environmental behavior, and satisfaction of local government pro-environmental behavior (57). Satisfaction of government pro-environmental behavior will be influenced by public participation in pro-environmental behavior, public environmental awareness, and public socioeconomic status (57). Meanwhile, public awareness and attention of environmental health issues and voluntarily participating in pro-environmental behavior both have a critical impact on pro-environmental and pollution control in a country or region. Li et al.'s (57) study finds that citizen participation can help minimize the information asymmetry in government pro-environmental work. After citizens acquire more information about environmental issues through participation pro-environmental activities, they will have a more in-depth understanding of environmental pollution issues, and their satisfaction will be leveled up because of their lower expectation rising from their empathy toward government pro-environmental work.

### The reverse moderating effect of household income

From the perspective of individual characteristics, studies have shown that household income, pro-environmental knowledge, and environmental pollution perception variables all have a positive impact on personal pro-environmental behavior (58, 59). When residents' income level is relatively low, they may focus more on needs at the survival level, and this survival level corresponds to their cognitive abilities. The requirement for environmental quality belongs to the safety needs level in Maslow's hierarchy of needs theory (60), while environmental participation is at the satisfaction of self-realization needs level, and only individuals with higher social status are likely to pay attention to environmental health information related issues. Among affluent regions and upper middle classes, individuals present stronger environmental awareness (61, 62). Due to this, they are more likely to acknowledge the negative impact of environmental pollution on their quality of life, and therefore they have higher standards for the timeliness and content adequacy of the government's environmental health information disclosure. Individuals with higher education and income level demonstrate a higher probability of participating in pro-environmental behavior, hence an individual's social and economic status also have an indirect impact on their satisfaction with government's pro-environmental behavior, at the same time the higher the individual income level, the more attention the individual will put on the quality of life and the long-term development (57).

**Figure 1:**
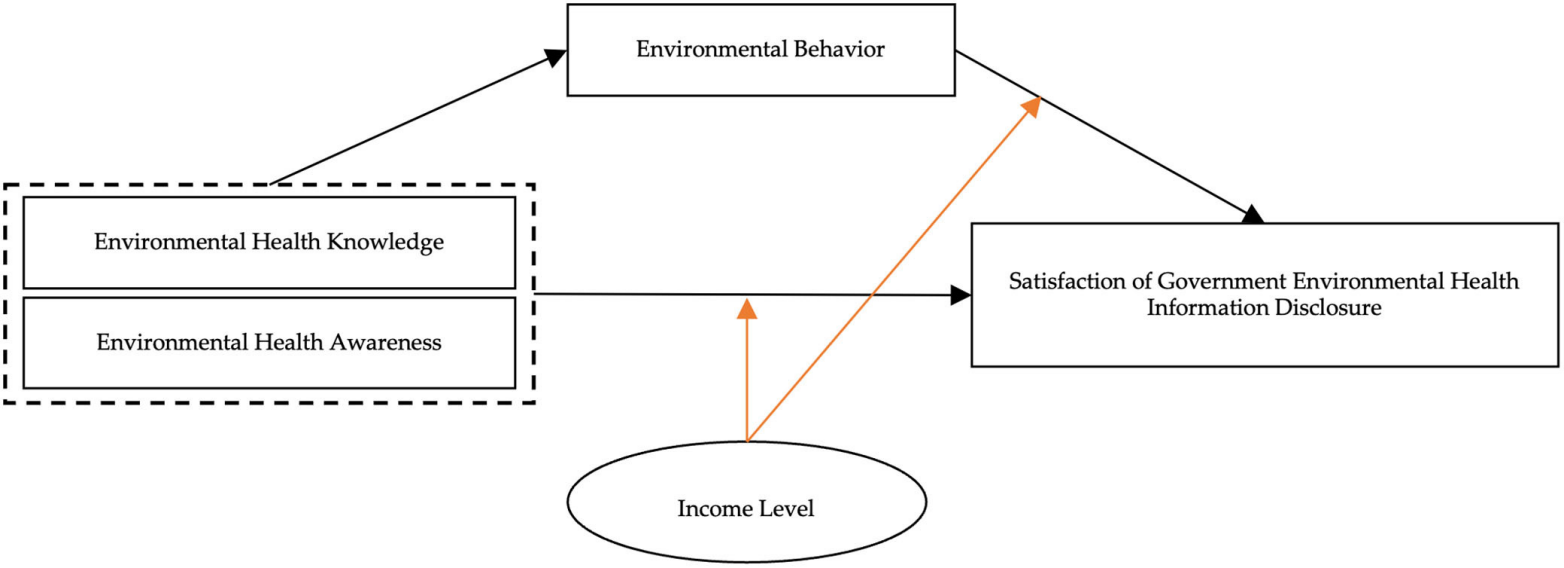
Proposed concept model.

Based on the above context, this paper brings up three hypotheses:

H1. environmental health knowledge and environmental health awareness have a positive effect on satisfaction of government environmental health information disclosure (The effect is showed in Figure 1).

H2. environmental health knowledge and environmental health awareness affect satisfaction of government environmental health information disclosure through the mediating effect of individual environmental behavior (The mediating effect is showed in Figure 1).

H3. environmental health knowledge and environmental health awareness affect satisfaction of government environmental health information disclosure through the reverse moderating effect of household (The moderating effect is showed in Figure 1).

## Method

### Satisfaction survey research

Satisfaction survey research originated from the customer satisfaction survey in retail and management industry, which was brought up by psychologists in the 1980s and adopted by enterprises in the 1990s, then gradually adopted by sociology, environmental management, economics, and other disciplines (63–65). A satisfaction survey is commonly used to measure the quality of a particular product or service by analyzing interviewees' expectations and actual feelings toward the product or service through questionnaires (63, 64, 66). By adopting the satisfaction survey, public perspective of the measured question can be obtained while minimizing the individual differences. This paper adopts the satisfaction survey to collect data from the research subjects. Within the 1,706 persons we surveyed, after removing the questionnaires which contained missing and unusual data, 717 respondents who are University students were selected for our research.

### Questionnaire

This paper adopts the Likert-scale questionnaire (67) which consists of five sets of questions including satisfaction of government environmental health information disclosure, environmental health knowledge, environmental health awareness, self-reported environmental behavior, and demographic factors. Answers to the questions in the questionnaire are scored from 1 to 5, after reverse scoring respondents' answers, scores of the first four sets of questions are added separately to obtain four total scores to represent the matching variables.

Satisfaction of government environmental health information disclosure (gehid) is evaluated by 4 questions including satisfaction of timeliness (geidtime) and content adequacy (geidcon) of general environmental health information and timeliness (ehidtime) and content adequacy (ehidcon) of environmental health information disclosure. Questions are as follows, “Are you satisfied with the timeliness of disclosure of environmental emergencies (for example COVID-19 pandemic)?” “Are you satisfied with the content of environmental emergencies (for example COVID-19 pandemic)?” “Are you satisfied with the timeliness of the air quality information disclosure?” and “Are you satisfied with the content adequacy of the impact of smog on human health publicized by the government?”.

Environmental health knowledge (ehk) contains 6 questions from three aspects, environmental knowledge, health knowledge, and environment health knowledge. Environmental knowledge is assessed by asking questions such as “Domestic waste segregation would benefit resource recycling” and “Haze is mainly caused by fine particulate matter (such as PM2.5).” While health knowledge is evaluated through questions like “Exercise helps to boost your immune system” and “The typical symptoms of COVID-19 are fever, fatigue, dry cough, and dyspnea.” Moreover, environmental health knowledge is appraised by questions such as “Land and groundwater which were easily polluted by toxic waste could affect human health” and “Eating wild animals may lead to infectious diseases.”

Environmental health awareness (eha) contains 5 questions. Environmental awareness is assessed through questions like “Have you paid attention to smog issues since the pandemic?” and “Have you paid attention to legal cases of illegally discharging of pollutants in this city and the penalties?”. Meanwhile, health awareness is evaluated by the following questions, “Can you ensure you took a comprehensive health check at least once a year?”, “Have you paid attention to the impact of haze on your health since the pandemic?” and “Do you know you can call 12369 to make a compliant when facing environmental pollution's negative effects on human health?”.

Self-reported environmental behavior (eb) is measured by the following 2 questions, “Have you considered taking protective measures (such as wearing a mask) when government announced severe haze pollution since COVID-19 pandemic?” and “On a daily basis, how long do you usually spend on browsing environmental health information since COVID-19 pandemic?”.

Demographic factors contain gender, age, education level (education), family size (family), monthly salary (salary) and apartment size (apart). As for gender (Male = 1, Female = 0), out of the 717 participants, 286 were males, accounting for about 40%, and 431 were females, accounting for about 60%^1^. Age is measured at six different age groups (18–24 = 1, 25–29 = 2, 30–39 = 3, 40–49 = 4, 50–59 = 5, 60 and above = 6), and education level is measured at six scales (Elementary school and below = 1, Middle school = 2, High school/Secondary school = 3, Junior University = 4, Undergraduate = 5, Postgraduate and above = 6), while family size is evaluated at five degrees (1-member = 1, 2-members = 2, 3-members = 3, 4-members = 4, above 4-members = 5). Self-reported monthly salary is assessed at five levels, 30.68% respondents' monthly salary are under 2,000 RMB (about 290 USD^2^), 28.17% are between 2,000 and 5,000 RMB (about 290–725 USD), 11.02% are between 5,000 and 8,000 RMB (about 725–1,159 USD), 11.02% are between 8,000 and 10,000 RMB (about 1,159–1,449 USD), and 13.39% are 10,000 RMB (about 1,449 USD) and above. Apartment size is another key indicator reflecting household income level, the results present that bungalow apartments with shared kitchen and bathroom (about 20 m^2^/215 f^2^) account for 34.45%, one-bedroom apartments (about 60 m^2^/646 f^2^) account for 1.53%, two-bedroom apartments (about 90 m^2^/969 f^2^) account for 19.8%, three-bedroom apartments (about 130 m^2^/1,399 f^2^) account for 31.24%, while four-bedroom apartments (about 160 m^2^/1,722 f^2^) and above account for 12.97%. As respondents tend to choose the salary that is lower than their actual salary in questionnaire, instead of using monthly salary to represent sample's household income level, apartment size is adopted to represent income level of the sample in our regression.

### Questionnaire reliability and validity test

Questionnaire reliability test examines the heterogeneity, stability, and reliability of scale valuation, and it is often evaluated by the internal consistency coefficient Cronbach's Alpha. The overall reliability coefficient of the questionnaire is 0.799, suggesting that consistency is “acceptable.” The questionnaire validity test is assessed through Kaiser-Meyer-Olkin (KMO) test and Bartlett's test of sphericity, and it is found that the KMO value is 0.82, *p* = 0.000 (KMO > 0.7, Bartlett's test of sphericity value *p* < 0.05), and hence the questionnaire passes the validity test.

## Results and analysis

### Descriptive statistics

According to the descriptive statistical in Tables 2, 3, frequency of respondents' gehid scores at 12, 14, and 16 are relatively high, with 14 representing the highest percentage of frequency. The average gehid score during the pandemic is 13.89, which represents that sample's satisfaction of government environmental health information disclosure during the pandemic is 13.89. While the maximum value is 20, and the minimum value is 4, and therefore it can be concluded that respondents' satisfaction is slightly higher than the “generally satisfied” level, yet there is still a huge gap between the current level and the “very satisfied” level. To be more specific, the average values of timeliness and content adequacy of the satisfaction of general health information disclosure are 3.51 and 3.60, respectively, and for the satisfaction of environmental health information disclosure the values are 3.35 and 3.42, respectively, which shows respondents have relatively lower satisfaction in respect of timeliness and content adequacy of government environmental health information disclosure compared to general health information disclosure. Overall, respondents' satisfaction barely passes the “generally satisfied” level, and the fact that respondents' satisfaction is not close to the “very satisfied” level also indicates quality of the government's environmental health information disclosure during the pandemic could be enhanced, and further improvements are required in terms of advancing the timeliness and content adequacy of the government's environmental health information disclosure. Moreover, the frequency distribution of ehk is left skewed, representing that University students' environmental health knowledge is at a relatively high level. The average score of ehk is 24.64, higher than the average level 18. For environmental health awareness, frequency of respondents' eha scores at 13 and 14 are relatively high, suggesting that University students' environmental health awareness is not as good as their environmental health knowledge. The average score of eha is 13.98, marginally lower than the average score 15. Furthermore, frequency of respondents' eb scores at 6, 7, 8, and 9 are comparatively high, demonstrating that University students regularly practice pro-environmental behavior. University students' average scores of environmental behavior is 7.31, which is higher than the average level 6. Other descriptive statistical information of variables can be found in Tables 2, 3.

**Table 2:** Frequency distribution of kernel variables.

	**Value**	**Frequency**	**Percent**	**Cumulative percent**
gehid	4	3	0.42	0.42
	5	3	0.42	0.84
	6	7	0.98	1.81
	7	4	0.56	2.37
	8	20	2.79	5.16
	9	17	2.37	7.53
	10	34	4.74	12.27
	11	35	4.88	17.15
	12	114	15.9	33.05
	13	73	10.18	43.24
	14	123	17.15	60.39
	15	60	8.37	68.76
	16	122	17.02	85.77
	17	19	2.65	88.42
	18	23	3.21	91.63
	19	7	0.98	92.61
	20	53	7.39	100
ehk	6	1	0.14	0.14
	12	1	0.14	0.28
	13	2	0.28	0.56
	14	1	0.14	0.7
	15	4	0.56	1.26
	16	3	0.42	1.67
	17	6	0.84	2.51
	18	30	4.18	6.69
	19	31	4.32	11.02
	20	33	4.6	15.62
	21	40	5.58	21.2
	22	54	7.53	28.73
	23	50	6.97	35.7
	24	71	9.9	45.61
	25	64	8.93	54.53
	26	63	8.79	63.32
	27	66	9.21	72.52
	28	69	9.62	82.15
	29	58	8.09	90.24
	30	70	9.76	100
eha	5	6	0.84	0.84
	6	5	0.7	1.53
	7	13	1.81	3.35
	8	27	3.77	7.11
	9	46	6.42	13.53
	10	56	7.81	21.34
	11	57	7.95	29.29
	12	70	9.76	39.05
	13	79	11.02	50.07
	14	89	12.41	62.48
	15	58	8.09	70.57
	16	40	5.58	76.15
	17	38	5.3	81.45
	18	32	4.46	85.91
	19	23	3.21	89.12
	20	24	3.35	92.47
	21	18	2.51	94.98
	22	10	1.39	96.37
	23	6	0.84	97.21
	24	7	0.98	98.19
	25	13	1.81	100
eb	2	9	1.26	1.26
	3	15	2.09	3.35
	4	28	3.91	7.25
	5	46	6.42	13.67
	6	121	16.88	30.54
	7	137	19.11	49.65
	8	170	23.71	73.36
	9	119	16.6	89.96
	10	72	10.04	100

**Table 3:** Descriptive statistics.

**Variables**	**Mean**	**Standard error**	**Minimum**	**Maximum**
gehid	13.89	3.100	4	20
Geidtime	3.512	1.085	1	5
Geidcon	3.598	0.993	1	5
Ehidtime	3.351	0.828	1	5
Ehidcon	3.424	0.845	1	5
ehk	24.64	3.794	6	30
eha	13.89	4.11	5	25
eb	7.309	1.771	2	10
Salary	2.483	1.375	1	5
Apart	2.868	1.487	1	5
Gender	0.399	0.49	0	1
Age	1.124	0.374	1	3
Education	5.063	0.681	4	6
Family	3.789	0.823	1	5

### Environmental health knowledge

#### Direct effect and mediating effect

By applying a simple mediation model (Hayes PROCESS Model 4) (68), Table 4 indicates that in the relationship between environmental health knowledge and the satisfaction with the government's environmental health information disclosure, environmental health knowledge presents a significant prediction effect on the satisfaction of government environmental health information disclosure (*B* = 0.1334, *t* = 4.4748, *p* < 0.001). After adding the mediating variable, the direct prediction effect remains significant (*B* = 0.0286, *t* = 1.7402, *p* < 0.01). It is also found that environmental health knowledge displays a significant positive prediction effect on environmental behavior (*B* = 0.0625, *t* = 7.4021, *p* < 0.001) and the satisfaction with the government's environmental health information disclosure (*B* = 1.3362, *t* = 10.8972, *p* < 0.001). Additionally, the direct effect of environmental health knowledge and the mediating effect of environmental behavior both do not contain 0 at upper and lower limits of bootstrap 95% confidence interval (Table 5), which demonstrates that environmental health knowledge not only could directly affect the satisfaction with the government's environmental health information disclosure, but also could enhance satisfaction through the mediating effect of environmental behavior. Meanwhile, the result shows direct effect (0.0499) and mediating effect (0.0835) account for 37.41% and 62.59% in the total effect (0.1334) respectively (Table 5). Indicating that for environmental knowledge.

**Table 4:** Regression results.

**Outcome variables**	**Predictor variables**	***B***	***t***	***R^2^***	***F***
gehid	ehk	0.1334	4.4748***	0.0592	8.9511***
	Gender	0.4276	1.8054*		
	Age	0.6018	1.7891*		
	Education	-0.7284	−3.8601***		
	Family	0.2373	1.7262*		
eb	ehk	0.0625	7.4021***	0.0753	11.5731***
	Gender	0.0056	0.0839		
	Age	0.0405	0.4254		
	Education	-0.0927	−1.7332*		
	Family	-0.0308	−0.7911		
gehid	ehk	0.0286	1.7402*	0.194	28.4862***
	eb	1.3362	10.8972***		
	Gender	0.4201	1.9149*		
	Age	0.5476	1.7575*		
	Education	-0.6046	−3.4518***		
	Family	0.2785	2.1860**		

^***^, ^**^, and ^*^ indicate significance at the 1, 5, and 10% level, respectively.

**Table 5:** Total, direct, and indirect effect.

	**Effect**	**Boot SE**	**Boot CILL**	**Boot CIUL**	**Percent**
Total effect	0.1334	0.035	0.064	0.202	
Direct effect	0.0499	0.0332	−0.0169	0.1142	37.41%
Mediating effect	0.0835	0.0148	0.0557	0.1144	62.59%

Boot SE, Boot CILL, and Boot CIUL refer to standard error of indirect effect estimated by deviation-corrected percentile Bootstrap method, and lower and upper limits of 95% confidence interval, respectively.

#### Moderating effect of income level

By applying Hayes PROCESS Model 8 moderated mediation (68), Table 6 shows that after adding apartment size into the model, the product of environmental health knowledge and apartment size shows a significant prediction effect on the satisfaction of government environmental health information disclosure (*B* = −0.0611, *t* = −3.2257, *p* < 0.05). While the product of environmental behavior and apartment size presents a significant prediction effect on the level of satisfaction (*B* = −0.2767, *t* = −3.4658, *p* < 0.01), suggesting that income level not only demonstrates a moderating effect on the direct prediction of the impact of environmental health knowledge, but also adjusts the prediction of environmental behavior on satisfaction.

**Table 6:** Moderated mediation results.

**Outcome variables**	**Predictor variables**	***B***	***t***	***R^2^***	***F***
eb	ehk	0.0644	7.6215***	0.0866	9.6063***
	apart	−0.0649	−2.9635**		
	ehk*apart	−0.0009	−0.1523		
	control variables	Controlled	Controlled		
gehid	ehk	0.0466	1.6475*	0.2306	23.543***
	eb	1.3026	10.7788***		
	apart	−0.0889	−1.2538		
	ehk*apart	−0.0611	−3.2257**		
	eb*apart	−0.2767	−3.4658***		
	control variables	Controlled	Controlled		

^***^, ^**^, and ^*^ indicate significance at the 1, 5, and 10% level, respectively.

**Figure 2:**
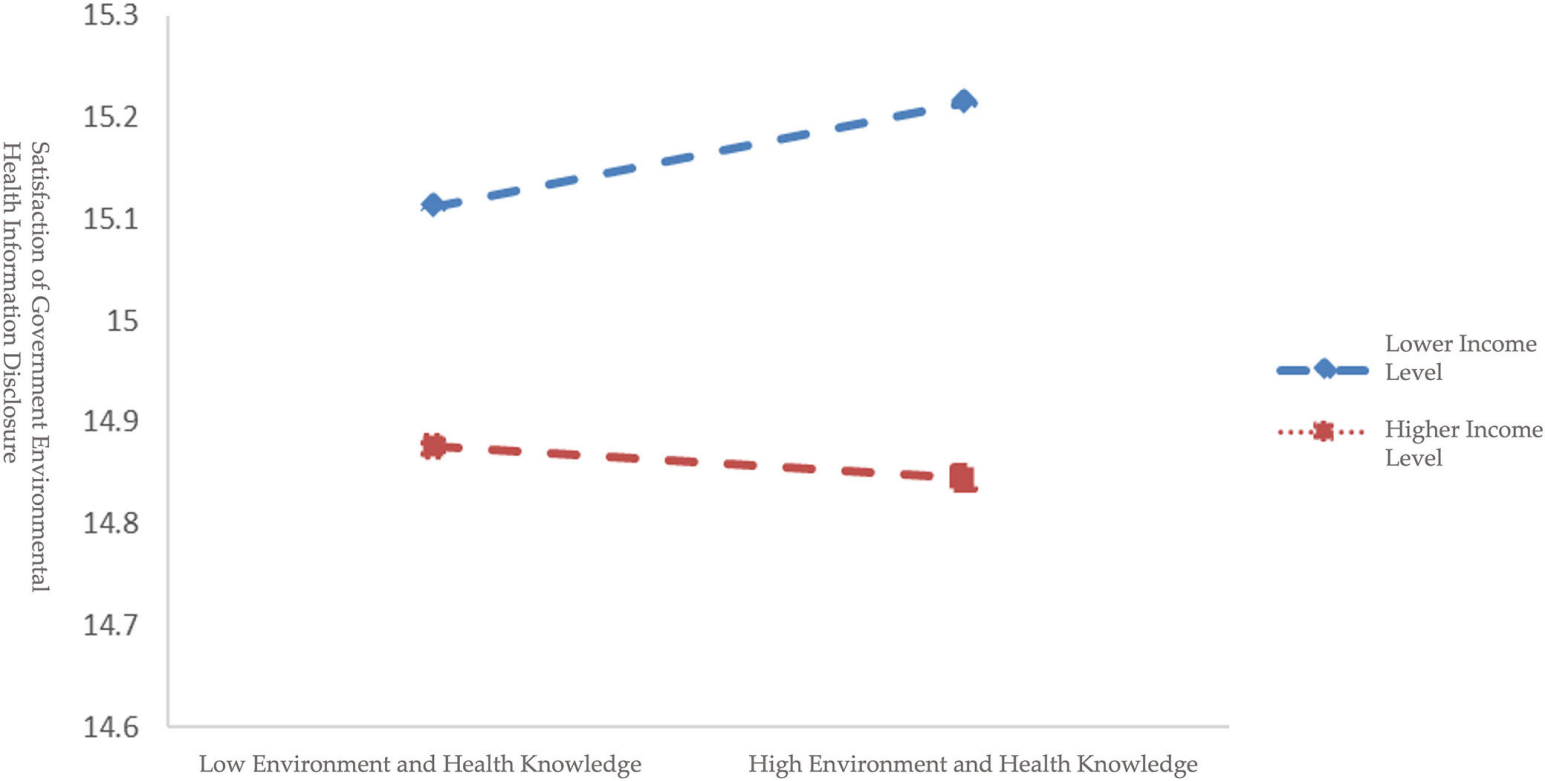
Moderating effect of income level between environmental health knowledge and satisfaction of government environmental health information disclosure.

**Figure 3:**
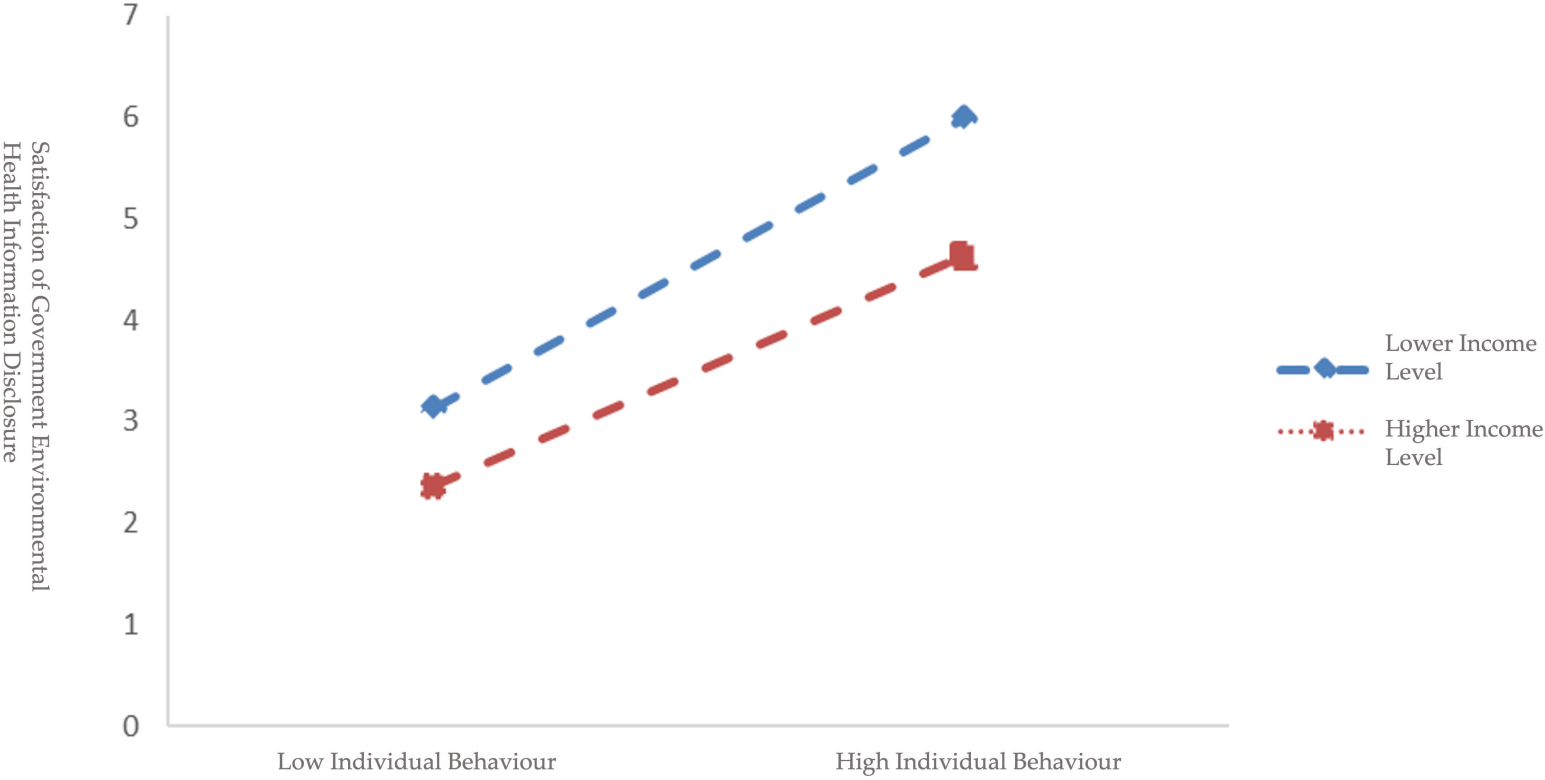
Moderating effect of income level between environ-mental health knowledge and satisfaction of government environmental health information disclosure.

Moreover, further simple slope analysis shows additional results among different income groups (Figures 2, 3). M-1SD (Table 7) represents the lower income level group with the mean being 1.38, representing respondents with bungalow apartments with shared kitchen and bathroom, and one-bedroom apartments, whose monthly salary is under 5,000 RMB (about 725 USD). While M+1SD (Table 7) represents higher income level group with mean being 4.35, indicating respondents with three-bedroom apartments and above, whose monthly salary is above 8,000 RMB (about 1,159 USD). According to Figure 2, environmental health knowledge represents a significant positive prediction effect on the satisfaction for respondents with a lower income level (M-1SD) with simple slope being 0.1374 (*t* = 3.5492, *p* < 0.001), and for those with a higher income level (M+1SD) environmental health knowledge presents no significant effect with simple slope being −0.0443 (*t* = −1.0790, *p* > 0.1), indicating that as income level increases the impact of environmental health knowledge on satisfaction displays a trend that decreased continuously until it disappeared. Based on Figure 3, for respondents with a lower income level (M-1SD) environmental behavior shows a significant positive prediction effect with simple slope being 1.7140 (*t* = 10.2628, *p* < 0.001). As for the higher income level (M+1SD) respondents, environmental behavior remains a significant positive prediction effect with simple slope being 0.8911 (*t* = 5.1885, *p* < 0.001), which demonstrates that with the increasing income level, the positive prediction effect of individual behavior on satisfaction has declined with a gradually decreasing trend. In addition, at three income levels, the mediating effect of individual behavior in the relationship between environmental health knowledge and satisfaction also shows a declined trend (Table 7), that is, as respondents' income level increased, it is more difficult to increase respondents' satisfaction through environmental behavior.

**Table 7:** Mediating effect at different income levels.

	**Income**	**Effect**	**Boot SE**	**Boot CILL**	**Boot CIUL**
Direct effect	eff1(M-1SD)	0.1374	0.0387	0.0614	0.2134
	eff2(M)	1.3026	0.0283	−0.0089	0.102
	eff3(M+1SD)	−0.0443	0.041	−0.1249	0.0363
Moderated mediation	eff1(M-1SD)	0.1125	0.0255	0.0653	0.1653
	eff2(M)	0.0839	0.0148	0.0557	0.1141
	eff3(M+1SD)	0.0562	0.0167	0.0271	0.0917
Moderated mediation comparison	eff2-eff1	−0.0287	0.0187	−0.0675	0.0056
	eff3-eff1	−0.0563	0.0306	−0.1183	0.0018
	eff3-eff2	−0.0276	0.0123	−0.051	−0.0027

M-1SD, M, and M+1SD refer to 1 standard deviation below the mean of income, mean of income, and 1 standard deviation above the mean of income from the mean, respectively.

### Environmental health awareness

#### Direct effect and mediating effect

Similarly, Table 8 indicates that environmental health awareness has a significant prediction effect on satisfaction (*B* = 0.4969, *t* = 11.5606, *p* < 0.01), and after adding the mediating variable, the direct prediction effect remains significant (*B* = 0.3248, *t* = 6.7411, *p* < 0.01). Furthermore, environmental health awareness presents a significant positive prediction effect on environmental behavior (*B* = 0.1831, *t* = 15.6196, *p* < 0.01), and environmental behavior demonstrates a significant positive prediction effect on satisfaction (*B* = 0.94, *t* = 7.0662, *p* < 0.01). Additionally, Table 9 displays that direct effect of environmental health awareness and mediating effect of environmental behavior do not include 0 at upper and lower limits of bootstrap 95% confidence interval, indicating that environmental health awareness not only could directly affect the satisfaction, but also can affect the satisfaction through the mediating effect of environmental behavior. Moreover, the test found that direct effect (0.3248) and mediating effect (0.1721) account for 65.37% and 34.63% of the total effect (0.4969), respectively.

**Table 8:** Regression results.

**Outcome variables**	**Predictor variables**	***B***	***t***	***R^2^***	***F***
gehid	eha	0.4969	11.5606***	0.1858	32.4445***
	Gender	−0.2236	−0.9844		
	Age	0.2517	0.7998		
	Education	−0.4265	−2.4069*		
	Family	0.2467	1.9291*		
eb	eha	0.1831	15.6196***	0.2585	49.5614***
	Gender	−0.2356	−3.8045***		
	Age	−0.0804	−0.9372		
	Education	0.0206	0.4257		
	Family	−0.0267	−0.7668		
gehid	eha	0.3248	6.7411***	0.2393	37.2195***
	eb	0.94	7.0662***		
	Gender	−0.0021	−0.0095		
	Age	0.3273	−0.0095		
	Education	−0.4458	−2.6008**		
	Family	0.2718	2.1966**		

^***^, ^**^, and ^*^ indicate significance at the 1, 5, and 10% level, respectively.

**Table 9:** Total, direct, and indirect effect.

	**Effect**	**Boot SE**	**Boot LLCI**	**Boot ULCI**	**Percent**
Total effect	0.4969	0.046	0.402	0.587	
Direct effect	0.3248	0.049	0.23	0.4203	65.37%
Mediating effect	0.1721	0.0278	0.1186	0.2271	34.63%

Boot SE, Boot CILL, and Boot CIUL refer to standard error of indirect effect estimated by deviation-corrected percentile Bootstrap method, and lower and upper limits of 95% confidence interval, respectively.

#### Moderating effect of income level

Likewise, Table 10 illustrates that after adding apartment size into the model, the product of environmental health awareness and apartment size shows no significant direct prediction effect on the satisfaction with the government's environmental health information disclosure (*B* = −0.0072, *t* = −0.9597). Yet, the product of environmental behavior and apartment size demonstrates a relatively significant prediction effect (*B* = −0.2791, *t* = −3.2442, *p* < 0.05), indicating that income level does not have a moderating effect in the direct prediction of environmental health awareness on the satisfaction but could adjust the prediction of environmental behavior on the satisfaction with the government's environmental health information disclosure.

**Table 10:** Moderated mediation results.

**Outcome variables**	**Predictor variables**	***B***	***t***	***R^2^***	***F***
eb	eha	0.1803	15.1811***	0.5108	35.7612***
	apart	−0.0233	−1.1815		
	eha*apart	−0.0072	−0.9597		
	Control variables	Controlled	Controlled		
gehid	eha	0.3055	6.3758***	0.5105	27.6836***
	eb	0.9304	7.0651***		
	apart	−0.0509	−0.7356		
	eha*apar	−0.0314	−1.0468		
	eb*apart	−0.2791	−3.2442**		
	Control variables	Controlled	Controlled		

^***^, ^**^, and ^*^ indicate significance at the 1, 5, and 10% level, respectively.

**Figure 4:**
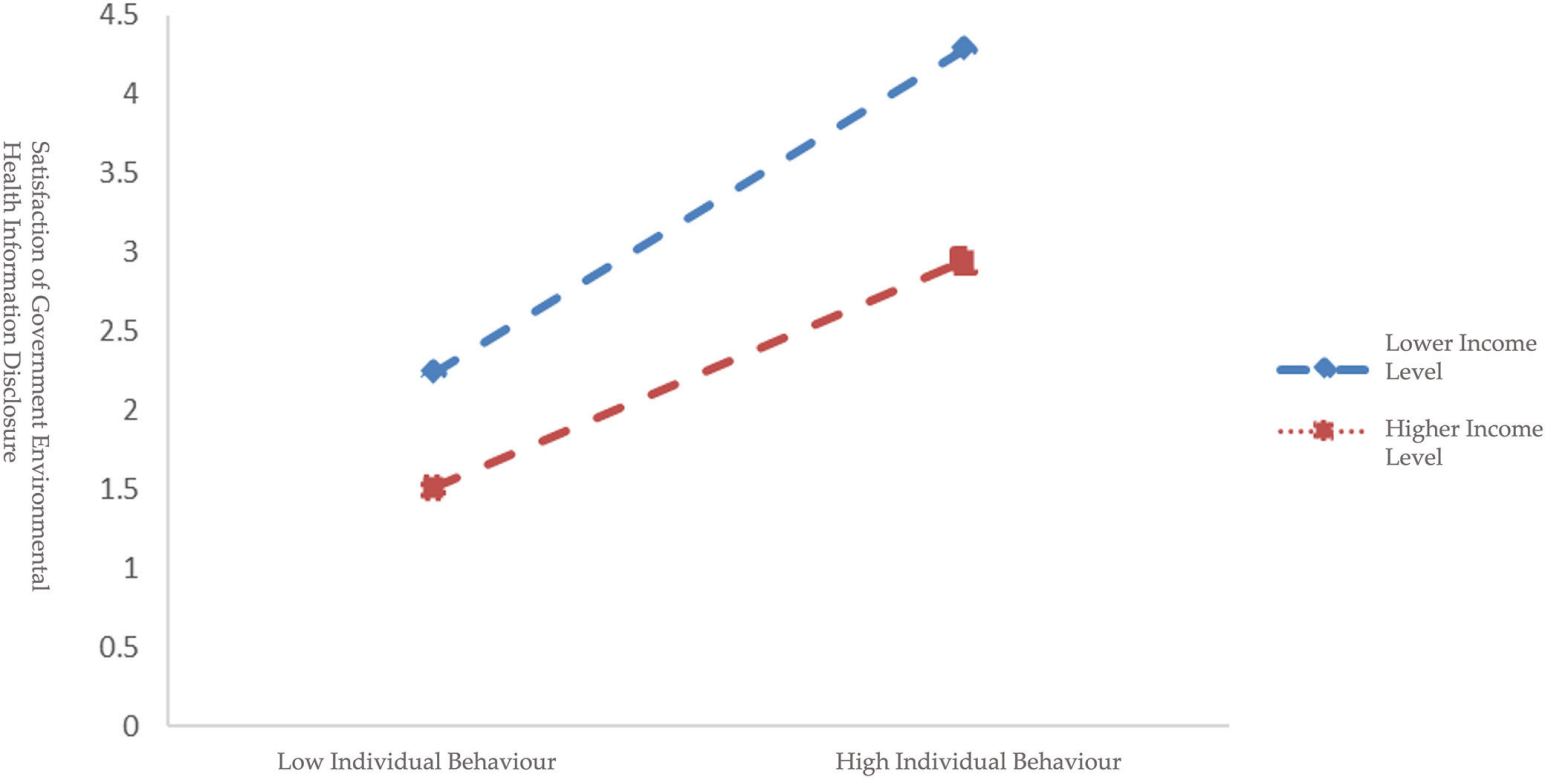
Moderating effect of income level between environmental behavior and satisfaction of government environmental health information disclosure.

Further simple slope analysis (Figure 4) demonstrates that environmental behavior of respondents with lower income levels (M-1SD) presents a significant positive prediction effect on satisfaction with simple slope being 1.3452 (*t* = 7.2461, *p* < 0.001). As for respondents with higher income levels (M+1SD), environmental behavior continues to have a significant positive prediction effect on satisfaction with simple slope being 0.5155 (*t* = 2.8408, *p* < 0.01), suggesting that as income level raises, the prediction effect of individual behavior on satisfaction is gradually declining. In addition, at three income levels, the mediating effect of individual behavior in the relationship between environmental health awareness and the satisfaction also displays a declining trend (Table 11), that is, as respondents' income increases, it is more difficult to enhance their satisfaction through environmental behavior.

**Table 11:** Mediating effect at different income levels.

	**Income**	**Effect**	**Boot SE**	**Boot LLCI**	**Boot ULCI**
Moderated mediation	eff1(M-1SD)	0.2568	0.0426	0.1785	0.3433
	eff2(M)	0.1677	0.0262	0.1187	0.2206
	eff3(M+1SD)	0.0874	0.0335	0.0235	0.1561
Moderated mediation comparison	eff2-eff1	−0.0891	0.0305	−0.1506	−0.0313
	eff3-eff1	−0.1694	0.0552	−0.2808	−0.0628
	eff3-eff2	−0.0803	0.0253	−0.1293	−0.0311

M-1SD, M, and M+1SD refer to 1 standard deviation below the mean of income, mean of income, and 1 standard deviation above the mean of income from the mean, respectively.

## Conclusions and policy recommendations

In sum, the overall quality of the government's environmental health information disclosure during the COVID-19 pandemic is relatively low according to our sample. In the past several year, China issued several laws and regulations regarding the government's environmental information disclosure, including Environmental Information Disclosure Measures (Trial) issued by the State Environmental Protection Administration in 2007, Regulations of the People's Republic of China on Disclosure of Government Information promulgated by the State Council of the People's Republic of China in 2008, Implementation Measures for Information Disclosure of Environmental Protection Public Institutions (for Trial Implementation) issued by the Ministry of Environmental Protection in 2010, and the revised Environmental Protection Law of the People's Republic of China in 2015, which gradually increased the quality of the government's environmental information disclosure system. Though the government disclosed environmental health related information during the COVID-19 pandemic, the overall quality, timeliness, and content adequacy still demand improvement for the government to be better prepared for the future public health crisis.

Second, satisfaction with the government's environmental health information disclosure is influenced by environmental health knowledge and awareness, and environmental behavior's significant positive mediating effect could be explained as the “unity of knowledge and action”. Therefore, by increasing individuals' environmental health knowledge and awareness, satisfaction will be enhanced both directly and indirectly. Research also indicates that public environmental awareness has shown an increasing trend since 2000 in China (69), and individuals present more active pro-environmental behavior in their private sphere as their environmental awareness increased over time (70). Therefore, it can be concluded that public environmental health knowledge and awareness have a positive impact on the quality of the government's environmental health information disclosure, while public environmental behavior shows substantial positive mediating effects among them.

Finally, the satisfaction with the government's environmental information disclosure differs among different household income levels, and household income level not only shows a moderating effect in the direct relationship between environmental health knowledge, awareness, and satisfaction with the government's environmental information disclosure, but also presents a moderating effect in the knowledge and awareness—environmental behavior—satisfaction relationship. As upper classes generally have better environmental cognition compared to that of lower classes (71), they quickly sense the negative impact environmental pollution brings to their lives, and therefore they require higher standards in the timeliness and content adequacy of the government's environmental health information disclosure. Similarly, a higher income level presents higher expectation of the quality of the environment, and therefore different household income levels demonstrate different satisfaction with the government's environmental health information disclosure.

According to the above conclusions, policy recommendations are addressed as follows. As the Chinese government has already set the goal of building a service-orientated government, the information disclosure function plays an essential role in achieving this goal. The government could build an environmental health information disclosure system to be better prepared for future public health crisis. According to our findings, different income groups demand different degrees of environmental health information, while the average income level in China has been increasing continuously. The public will require government to disclose higher quality environmental health information in the future. This sets the expectation for the government to construct an information disclosure framework at both national and local levels that connect all the existing different government departments' information disclosure systems. Thereby, government could be better prepared for the future public health crisis. To begin, the government could enhance the quality of the current environmental health information disclosure system by expanding the current mandatory disclosing information as departments rarely voluntarily disclose information (72). For instance, the government could require departments to disclose more environmental health information, especially the negative impact on human health to enhance citizens' environmental health knowledge and awareness. Moreover, the government should make sure of the timely delivery of environmental health information as currently some departments failed to disclose related information within the required 20 working days (72). For example, central government and local governments could set up online platforms at different levels for governments to disclose public health crisis related information and allow the public to easily access this information. With timely information, transmission of the virus could be reduced effectively, and citizen participation could be efficiently carried out through the online platforms as well. Healthcare institutions could adopt this methodology for disclosing related information to assist the reduction of transmission of the virus in future public health emergencies as well. As for content adequacy, the current government environmental health information disclosure system still demands expansion, especially environmental health information which has been proven essential in responding in a public health emergency. For example, the government could require related healthcare institutions to disclose not only negative environmental information but also the seriousness of the harmful impact on human health at an earlier stage in future public health crises, such as the transmission rate, the infection rate, the severity rate, and the case fatality rate of a certain virus. Citizens could then instantly identify the seriousness of certain information and decide their actions accordingly. However, the applicability of our conclusions to other social groups may be restricted due to the size of our research sample and selection bias in our questionnaire.

## Data availability statement

The raw data supporting the conclusions of this article will be made available by the authors, without undue reservation.

## Ethics statement

Ethical review and approval was not required for the study on human participants in accordance with the local legislation and institutional requirements. The patients/participants provided their written informed consent to participate in this study.

## Author contributions

RA: formal analysis, methodology, investigation, resources, data curation, writing—original draft preparation, validation, and visualization. FW and RA: writing—review and editing. FW: project administration. HY: software. KH and FW: conceptualization and funding. All authors contributed to the article and approved the submitted version.

## Funding

This research was funded by the Social Science Fund of Shaanxi Province (Grant No. 2020D030) and the Introduction of High-End Foreign Experts Project of the Ministry of Science and Technology of the People's Republic of China (Grant No. G2021041004L).

## Conflict of interest

The authors declare that the research was conducted in the absence of any commercial or financial relationships that could be construed as a potential conflict of interest.

## Publisher's note

All claims expressed in this article are solely those of the authors and do not necessarily represent those of their affiliated organizations, or those of the publisher, the editors and the reviewers. Any product that may be evaluated in this article, or claim that may be made by its manufacturer, is not guaranteed or endorsed by the publisher.

## Footnotes

^1^The percentage of male and female students at Northwest University in 2020 was 42.23% and 57.77% respectively.

^2^Converted using the average China/U.S. Foreign Exchange Rate in 2020 (1 RMB = 6.9 USD).
